# Meta-Analysis of Psychological Interventions for Reducing Stress, Anxiety, and Depression among University Students during the COVID-19 Pandemic

**DOI:** 10.3390/ijerph19159199

**Published:** 2022-07-27

**Authors:** Romualdas Malinauskas, Vilija Malinauskiene

**Affiliations:** Department of Physical and Social Education, Lithuanian Sports University, Sporto 6, LT-44221 Kaunas, Lithuania; vilija.malinauskiene@lsu.lt

**Keywords:** stress, anxiety, depression, university students, meta-analysis, COVID-19

## Abstract

(1) Background: The aim of this study is to investigate the effects of internet-based intervention programs for reducing stress, anxiety, and depression among university students during the COVID-19 pandemic by conducting a meta-analysis. (2) Methods: Searches were conducted in the following databases: MEDLINE, EbscoHost Academic Search Ultimate, and PsycArticles, using a combination of “Covid-19 AND ‘Randomized Controlled Trial’ AND students”, as well as a combination of the following search terms: “internet”, “online”, “treat_”, “psycholog_”, “intervention”, “program_”, “stress_”, “depress_”, “anxiety”, “university”, “college”, ”freshm_”, “sophomore_”, and “undergraduat_”. The population, intervention, control, outcomes, and study design (PICOS) framework was used (P (population): university students during the COVID-19 pandemic; I (intervention): internet-based intervention programs for reducing stress, anxiety, and depression; C (control): no intervention, usual care, or on a waiting list; O (outcomes): stress, anxiety, and depression indicators; S (study design): meta-analysis including only randomized controlled trials (RCTs)). A meta-analysis was performed on the 10 retrieved studies published between 2021 and 2022. Only RCTs were analyzed. (3) Results: All 10 analyzed papers revealed a trend in the effectiveness of internet-based intervention for reducing stress, anxiety, and depression in university students during COVID-19. Significant effects from the included RCTs with interventions for reducing stress and depression were established. (4) Conclusions: Psychological internet-based interventions may help to reduce depression and stress among university students; however, more research is needed to determine their effectiveness in reducing anxiety.

## 1. Introduction

Researchers have studied the causes, risks, and severity of stress, anxiety, and depression from various perspectives and across disciplines. Stress, anxiety, and depression are often analyzed because they are presently major mental health problems that cause disability globally [1]. Although the effects of anxiety, depression, and stress reduction programs in the context of the COVID-19 pandemic are frequently analyzed, there is still no answer to the question of which mental health problem these intervention programs are most effective for [2]. Additionally, studies on psychological interventions for students undergoing long-term, at-home quarantine in the context of the COVID-19 pandemic are limited. Previously conducted meta-analyses did not focus on university students but included other populations, such as healthcare workers, noninfectious chronic disease patients, COVID-19 patients, and quarantined persons [3] (p. 91). For instance, a meta-analysis involving 66 studies showed that during the COVID-19 pandemic, mental health problems, such as depression, anxiety, and stress, are common for different populations, especially healthcare workers, noninfectious chronic disease patients, COVID-19 patients, and quarantined persons. Overall, the pooled prevalence of depression, anxiety, and distress was 31.4%, 31.9%, and 41.1%, respectively [3] (p. 91). Another meta-analytical study assumed that 50% of students experience significant levels of stress, anxiety, and depression, suggesting that universities should apply preventive psychological interventions that could reach more students [4]. Furthermore, a meta-analysis comprising 84 examinations of 1,292,811 Chinese college undergraduates during the pandemic distinguished that more than one-fourth of Chinese college undergraduates have experienced depressive symptoms during quarantine [5]. Meta-analytical examinations have also revealed that pandemics and quarantine are mentally challenging periods for undergraduates [6] since stress, anxiety, and depression levels are considerably increased and can reach clinical levels in this population due to the COVID-19 pandemic [2].

Internet-based interventions were implemented before the pandemic because they have some advantages compared to face-to-face interventions: They are easily accessible to participants; they often involve only virtual instructors providing assistance in online formats (videos), who are not necessarily psychologists or therapists, allowing participants to remain anonymous; they are cost-effective, especially when they involve only some guidance [7]. However, evidence for the differences in the preferences of university students before the pandemic for internet-based psychological interventions versus face-to-face interventions is somewhat contradictory. For example, recent meta-analyses have suggested that students prefer guided self-help interventions to face-to-face interventions for depression, and these self-help interventions can have comparable effects and equal adherence compared to face-to-face interventions [7,8]. However, Benjet [9] found that students (hypothetically) prefer face-to-face interventions to internet-based (online) interventions, while Andrews et al. [10] found that online self-help interventions have low program completion rates, which may diminish the effectiveness of the intervention. Such differences could have occurred since one study focused on guided online self-help interventions, while the other on analyzed internet-based interventions, where health professionals, such as psychologists, public health nurses, psychotherapists, and program coaches, provided participants with support. In this study, we do not differentiate between internet-based interventions during the COVID-19 quarantine from this viewpoint.

Accordingly, an appraisal of the effectiveness of mental interventions in lessening stress, anxiety, and depression among college undergraduates in the pandemic context requires meta-analytical examination since, given the challenges related to this pandemic, college undergraduates are believed to be especially vulnerable to mental health issues, especially stress, anxiety, and depression [11]. Specifically, the psychological outcomes of quarantine during the COVID-19 pandemic have negatively impacted psychological health, and this impact can continue for some time [12].

College undergraduates usually experience different stressors, for example, academic stress, individual issues, career issues, and financial worries [13]. However, during the COVID-19 pandemic, undergraduates’ mental issues have only increased. They are now experiencing worsened depressive symptoms, diminished sleep quality, increased anxiety [14], social disconnectedness, an absence of peer support, loneliness, gloom, and outrage [15].

Many examinations have contended that health conditions during the COVID-19 pandemic might be connected to the hypothesis of “hypochondriac concerns”, which refers to = stress over the possibility of being infected by the disease [16]. Likewise, they might be connected to significant life changes during quarantine involving restrictions on movement, as well as the revocation of significant exercises, face-to-face interaction, and contact education [17]. To maintain accessible psychological health administration while lessening the probability of transmission of the virus, there has been a new drive to switch from regular face-to-face mental health treatments to online or telehealth treatments. Within this specific situation, there is an increased demand for effective internet-based and evidence-based mental interventions/programs/techniques that aid in addressing psychological health outcomes based on the information received from various pandemics [2,6]. Internet-based interventions can help college undergraduates and graduates adapt to stress, anxiety, and depressive symptoms and improve their mental health; they will also not need to commit to treatment [6]. Nevertheless, there are many types of internet-based interventions, but their effectiveness and scientific foundation are unclear. Subsequently, a review that could focus on mental interventions aimed at lessening stress, anxiety, and depression among college undergraduates and graduates in the pandemic context, as well as evaluate the effectiveness of such mental interventions, is required.

### This Study

In this study, we assume that internet-based intervention projects can contrastingly affect various psychological health issues, since a systematic survey and meta-analysis of stress management interventions for colleges students established that guided stress management interventions have moderate effects on stress and anxiety and small to moderate effects on depression [9,18].

Even though there are numerous studies on the effect of mental interventions on lessening anxiety, depression, and stress in individuals impacted by the COVID-19 pandemic [19], only a few have inspected the impacts of internet-based intervention programs on diminishing the stress, anxiety, and depression of college undergraduates and graduates during the COVID-19 pandemic. College students were an especially vulnerable population during the COVID-19 quarantine [20]. One of the first studies conducted in Italy concerning health risk perceptions related to COVID-19 and the effects of the quarantine experience on the psychological wellbeing of university students confirmed that university students represent a vulnerable population, and specific interventions are needed to protect their psychological wellbeing during the pandemic [20]. This study stipulated that “while the acute impact amidst COVID-19 quarantine seems clear (increase in scores; 42.5% for anxiety, 74.3% for depression, and 63.3% increase in total suicidal thoughts), the long-term consequences are unknown. However, the results constitute a clear message that vulnerable populations are at a need for specific interventions concerning their mental health issues” [21] (p. 2). Regardless, only distance intervention was possible during COVID-19 quarantine, and only internet-based interventions tested via randomized controlled trials (RCTs) can illustrate the evidence-based effectiveness of such types of interventions.

Coincidentally, not all reviews have utilized a control group or incorporated the required quantitative information (for example, descriptive statistics contrasting the intervention and the control group at baseline and posttest) (for an example, see [22]). This study is a meta-analysis determined to provide an evidence-based method of analyzing whether internet-based interventions can lessen stress, anxiety, and depression among college undergraduates and graduates during the COVID-19 pandemic. Furthermore, the population, intervention, control, outcomes, and study design (PICOS) framework [23] was adhered to (P (population): university students during the COVID-19 pandemic; I (intervention): internet-based intervention programs for reducing stress, anxiety, and depression; C (control): the control group received no intervention, received usual care, or were put on a waiting list; O (outcomes): stress, anxiety, and depression indicators; S (study design): meta-analyses including only RCTs).

## 2. Materials and Methods

### 2.1. Data Sources

**Figure 1:**
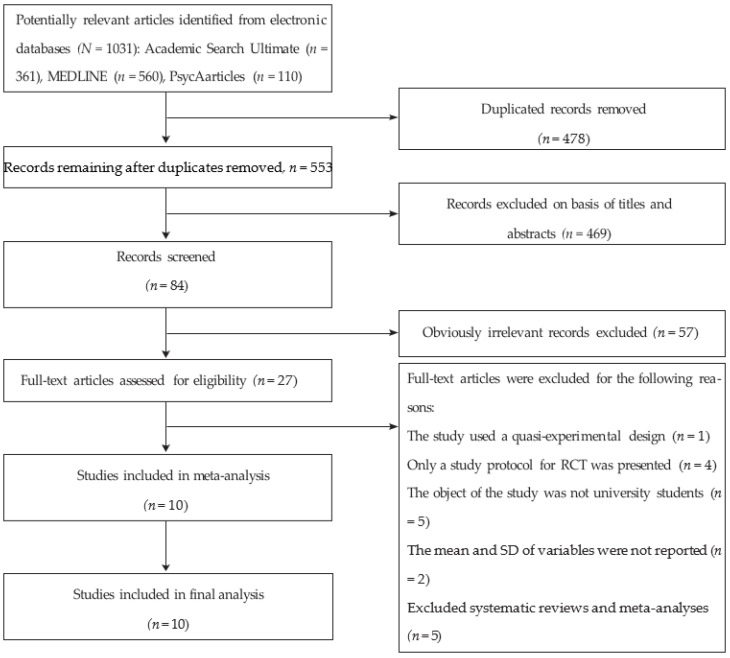
A flow diagram of studies included and excluded (per PRISMA recommendations).

This study was arranged using the preferred reporting items for systematic reviews and meta-analyses (PRISMA) proposals [24] (systematic review registration statement from INPLASY and registration number INPLASY202260054). Articles published in English in academic journals between 2021 and 2022 were searched for on MEDLINE, EbscoHost Academic Search Ultimate, and PsycArticles utilizing a combination of “COVID-19” AND “Randomized Controlled Trial” AND “students”, as well as a combination of the accompanying search terms: “internet”, “online”, “treat_”, “psycholog_”, “intervention”, “program_”, “stress_”, “depress_”, “anxiety”, “university”, “college”, ”freshm_”, “sophomore_”, and “undergraduat_”. Through this search technique, 1031 articles were found (Figure 1).

Subsequent to eliminating duplicates, two independent researchers screened the excess records’ titles and abstracts. The full text of each remaining article was then studied to evaluate whether it qualified for the study. Following this, supplementary reference analysis and manual searches were conducted to prevent qualifying studies from being disregarded. The final included articles were chosen through discussion. Information was extracted utilizing a standardized information extraction sheet. The accompanying information was also extracted: author/s, year of publication, study design, total sample size, participants’ details, control conditions, intervention attributes, intervention provider, result criteria (stress, anxiety, and depression diagnostic instruments), and study results.

### 2.2. Study Selection

Study selection involved choosing studies on interventions for diminishing stress, anxiety, and depression among college undergraduates and graduates in the pandemic context. Inclusion criteria for the current analysis were as follows: (1) journal articles and “un-published” Ph.D. dissertations that gave a quantitative assessment of the viability of the intervention for reducing stress, anxiety, and depression among college undergraduates and graduates in the pandemic context; (2) studies targeting college undergraduates and graduates aged 18 years or older during COVID-19; (3) studies with publication dates between 2020 and 2022 since COVID-19 restrictions started in 2020 and continued until 2022; (4) full-text studies published exclusively in English. Excluded from the review selection list were: (1) studies analyzing internet-based mental interventions that did not allude to the COVID-19 pandemic; (2) studies with their principal text not published in English (for example, just the tables were in English); (3) studies that did not incorporate signs of stress and/or anxiety and/or depression; (4) studies that examined internet-based mental interventions that did not have a decrease of stress and/or anxiety and/or depression as their essential intervention focus.

### 2.3. Study Inclusion

The included investigations for the meta-analysis needed to meet the accompanying inclusion criteria: (1) study design was restricted to RCTs; (2) the control group received no intervention, received usual care, or were put on a waiting list; (3) the review objective was to assess the impact of intervention programs on lessening stress, anxiety, and depression among college undergraduates and graduates during the COVID-19 pandemic; and (4) the accessible information of each study included an estimation of impact sizes.

The review was rejected if it did not satisfy the following accompanying criteria: (1) the review was a one-group pre-/post-comparison review; (2) the review utilized a quasi-experimental or non-equivalent control group pretest/posttest design; (3) the full text of the review was inaccessible; and (4) the review did not give adequate information (means and standard deviations (SDs) of stress and/or anxiety and/or depression were not detailed). According to these criteria, studies were chosen for meta-analysis.

### 2.4. Data Extraction and Quality Assessment

To identify the impact sizes of the treatment interventions, the sample sizes of the review (experimental) and control groups, as well as the means and SDs of stress, anxiety, and depression pretest and posttest, were coded as dependent factors. The interventional models were deciphered as a type of internet-based intervention. Studies were then analyzed similarly for the type of control (waiting list, no treatment, treatment as usual). The two authors extracted this information. The authors (raters) coded the information independently, and conflicts between the raters were settled via consensus. The interrater agreement between the two raters on the included studies was satisfactory (>80%).

The methodological quality of data was evaluated in this review using a scale based on criteria that the American Psychological Association created for evaluating empirically validated interventions [25,26]. The six standards used to evaluate methodological rigor were: (1) the randomization of the sample; (2) a comparison with different medications, standard administrations, or waiting list control; (3) the meaning of the population; (4) the utilization of approved and dependable result criteria (criteria for stress, anxiety, and depression; (5) the utilization of treatment manuals or curricula; and (6) a large sample size (i.e., more than 25 participants per group). One point was given for the presence of each standard. Consequently, each study recieved a score of 1 to 6, with higher scores showing higher levels of methodological quality. The two authors independently rated each of the six studies. To evaluate interrater reliability, Cohen’s kappa coefficient was utilized (the kappa coefficient was 1.00). The quality scores are shown in Table 1.

### 2.5. Outcome Measurement

The focus of the current review was on the three psychological health results of stress, anxiety, and depression, and self-administered evaluation tools were utilized to assess these. The review included in the meta-analysis utilized different survey scales: the Perceived Stress Scale (PSS-10); Warwick–Edinburgh Mental Wellbeing Scale (WEMWBS); Patient Health Questionnaire (PHQ-4)—a brief survey of anxiety and depression estimation; Depression, Anxiety, and Stress Scales (DASS-21); PHQ-9 for depression estimation; Beck Anxiety Inventory (BAI); Beck Depression Inventory-II (BDI-II); Online Test Anxiety Inventory (OTAI); Short Health Anxiety Inventory (SHAI); Patient-Reported Outcome Measurement Information System’s (PROMIS) anxiety and depression scales; and the Generalized Anxiety Disorder Screener (GAD-7).

### 2.6. Data Analysis

For the studies that utilized an RCT design, the intervention group comprised all participants receiving mental treatment, and the control group comprised all participants not receiving this treatment, receiving treatment as usual, or being on a waiting list. The results’ mean values and SDs were recorded before and after the intervention for both the experimental and control groups. We analyzed pre- to post-intervention changes to evaluate the impacts of mental interventions; however, we did not analyze follow-up impacts because of a lack of follow-up information in a few of the studies and because the follow-up periods varied. Result measure changes were then used to assess the impact size of the effectiveness of the mental intervention. Impact size was determined for each recorded result (stress, depression, and anxiety) utilizing means and SDs to estimate the standardized mean difference (SMD, Hedges’ g).

For every indicator of psychological health, the pooled estimates (SMD) and 95% confidence intervals (CI) of impact sizes were determined using an inverse-variance weighted random-impacts meta-analysis model [27]. The *I^2^* statistic was utilized to evaluate irregularity (heterogeneity) across studies included in the meta-analysis, with values greater than 50% indicating high irregularity [28]. To test for heterogeneity, we determined Cochran’s Q-statistic, which considers the degrees of freedom. For the null theory (which posits that all impact sizes are equivalent) to be dismissed, Cochran’s Q-statistic had to be statistically significant, and the extent of the error variance among the complete difference observed from the impact sizes had to be significantly high given sampling errors [29]. The degree of heterogeneity was evaluated using the equation: *I^2^* = 100% × (Q − (k − 1))/Q, where *k* represents the number of studies included. The *I^2^* statistic values of 25%, 50%, and 75% showed low, moderate, and high degrees of heterogeneity, respectively.

We collected included in meta-analysis studies as indicated by the intervention’s impacts on the different mental health issues, i.e., stress, depression, and anxiety, and the subgroup of meta-analysis was directed. All meta-analyses were performed with R statistical package Meta-Analysis via Shiny (MAVIS) R-Shiny software [30]. To investigate conceivable publication predisposition, we created funnel plots using MAVIS R-Shiny software. In asymmetry conditions (if any), Duval and Tweedie’s Trim and Fill strategy [31] was utilized to estimate the modified pooled impact size.

## 3. Results

### 3.1. Characteristics of the Included Studies

Ten studies were found that met our inclusion criteria. All incorporated studies assessed the effectiveness of internet-based psychological intervention programs for reducing stress, anxiety, and depression among university students during COVID-19 social distancing. Seven studies were conducted by researchers in American universities [32,33,34,35,36,37,38], two studies in Asian universities [39,40], and one study in an African university [41]. Meta-analyses were performed separately on interventions for reducing stress, anxiety, and depression (Table 1). Additionally, 10 analyzed papers were RCTs. Studies using a quasi-experimental pretest/posttest design were not analyzed in the present study. Incorporated into the meta-analyses were internet-based interventions, which were categorized as brief online Isha Upa yoga, centering meditation interventions, positive psychotherapy, cognitive-behavioral therapy (CBT), mindfulness-based interventions (MBI), and breathing training programs. The types of interventions of each study are presented in Table 1.

A total of 5086 students 18 years or older participated in the studies. Three of the studies reported an age range of participants [37,39,41], while the others just reported that students were 18 years or older. Most of the included studies were conducted with undergraduate students, while one study [39] featured graduate students. Finally, one study targeted only healthcare students [41], while the others targeted students from various study programs

### 3.2. Description of the Interventions Used in the Included Studies

The details of the interventions are summarized in Table 1. The number of sessions varied greatly, ranging from four [38] to forty [33]. The average number of sessions of the included mental interventions was 15.3 sessions, while the length of sessions varied between 10 and 90 min. The duration of the interventions also varied greatly, ranging from three weeks [40] to twelve weeks [32,35].

All 10 studies mentioned intervention providers (through online formats), which were categorized as follows: Isha Hatha yoga teacher [32]; college counselor [33]; instructor [34,41]; expert [40]; psychologist [35,39]; mindfulness teacher [37]; multidisciplinary team, including a study member and a mindfulness teacher, for the breathing application [36]; and a multidisciplinary team, including a psychologist and a mindfulness teacher [38]. Further, all 10 studies used internet-based/video-based interventions.

The results of all included studies except one [34] revealed that internet-based psychological interventions are effective for reducing mental health problems (anxiety, depression, stress) among college undergraduates and graduates in the pandemic context. However, in one RCT [34], individuals in the study group were treated with eight 12 sessions of 10 min each (for four weeks) using a self-help, internet-based positive psychology and CBT intervention, while participants in the control group received remote counseling services (as usual). However, anxiety levels did not differ between the study and control groups at baseline, after the intervention, or at the three-month follow-up. Additionally, in predicting anxiety, there were no significant interactions from the condition and pretreatment to posttreatment time [34].

### 3.3. The Effects of Internet-Based Psychological Interventions on Stress

Six randomized controlled trials noted the mean and SD of stress scores in the experimental and control groups posttest. The statistical indicators (mean and SD) of stress among university students during the COVID-19 pandemic in the RCTs included in the meta-analysis are presented in Table 2.

**Figure 2:**
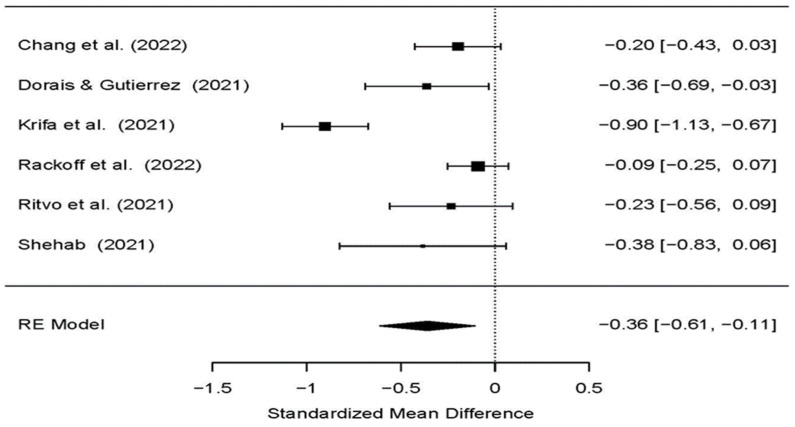
The effects of psychological interventions on reducing stress among university students during COVID-19 in the included randomized controlled trials [32,33,34,35,36,41].

A meta-analysis of the six RCTs reported a significant reduction in stress, showing that at posttest, the participants of the study (treatment) group, who were provided with internet-based psychological intervention, had significantly lower stress (*p* = 0.01) than those in the control group (SMD: −0.36; 95% CI [−0.61, −0.11]) (Table 3 and Figure 2).

For this group of studies, the random effects model was used because statistically significant heterogeneity was observed (*I^2^* = 81.99%; *p* < 0.001; Q = 34.19). The intervention with a large effect size for the reduction of stress was the internet-based positive psychology intervention program (SMD: −0.90; 95% CI: −1.13 to −0.67), (Figure 2).

### 3.4. The Effects of Internet-Based Psychological Interventions on Anxiety

**Figure 3:**
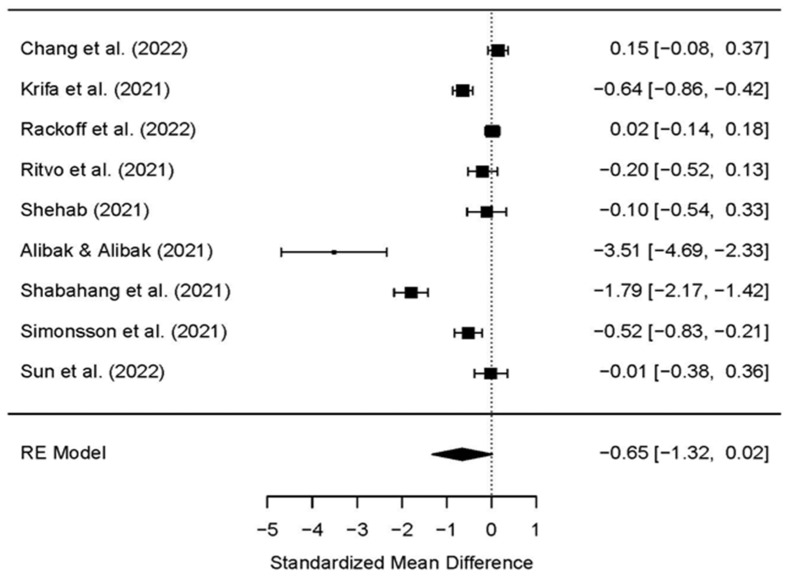
The effects of psychological interventions on reducing anxiety among university students during COVID-19 in the included randomized controlled trials [32,34,35,36,37,38,39,40,41].

Nine RCTs, which contributed to a pooled analysis of internet-based psychological interventions on anxiety, showed no significant difference between the study (treatment) group and control group posttest (SMD: −0.65; 95% CI [−1.32, 0.02]) (Table 3 and Figure 3). High heterogeneity was established in anxiety indicator estimates, effect sizes across studies differed considerably (*I^2^* = 97.86%, *p* < 0.001; Q = 131.78), and the random effect model was used. The findings of data analysis suggest that there was a reduction in anxiety, with a standardized difference in the mean point estimate of −0.65 but this was not statistically significant (*p* = 0.06); further, the 95% CI was [−1.32, 0.02] for the SMD.

### 3.5. The Effects of Internet-Based Psychological Interventions on Depression

**Figure 4:**
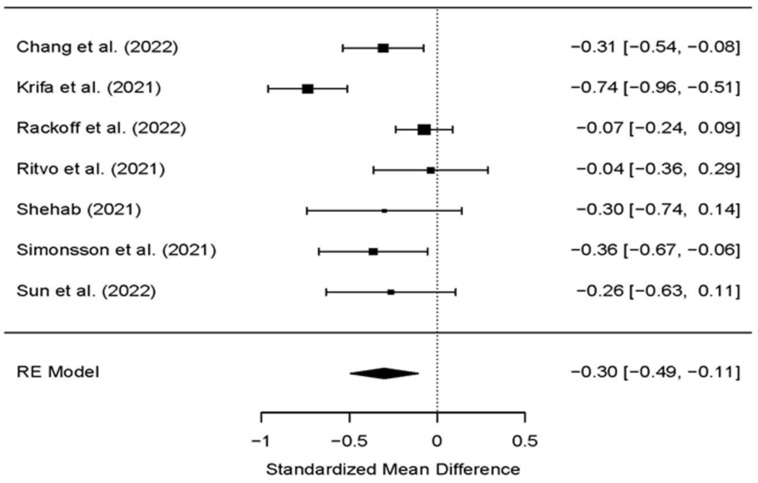
The effects of psychological interventions on reducing depression among university students during COVID-19 in the included randomized controlled trials [32,34,35,36,37,38,41].

Seven studies, which added to a pooled examination of internet-based mental interventions for depression, revealed that participants of the study (treatment) group who received an internet-based mental intervention had fundamentally lower (*p* = 0.00) depression than those in the control group posttest (SMD: −0.30; 95% CI: −0.49 to −0.11) (Table 3, Figure 4). Statistically significant moderate heterogeneity as evaluated using *I^2^* was noticed (*I^2^* = 71.46%; *p* < 0.001; Q = 24.46), so the random impact model was utilized. The intervention with a large effect size for decreasing depression was the internet-based positive psychology intervention program (SMD: −0.74; 95% CI: −0.96 to −0.51) (Figure 4) [41].

**Figure 5:**
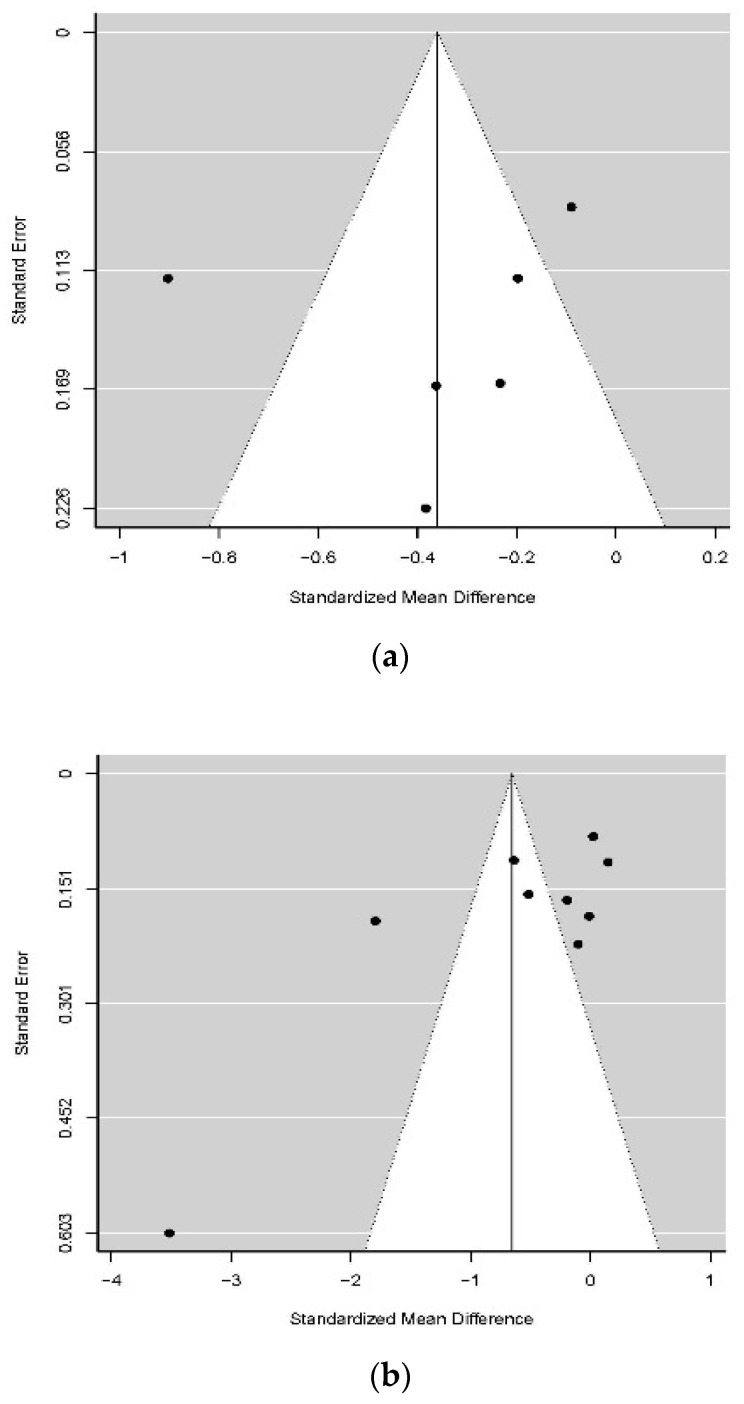

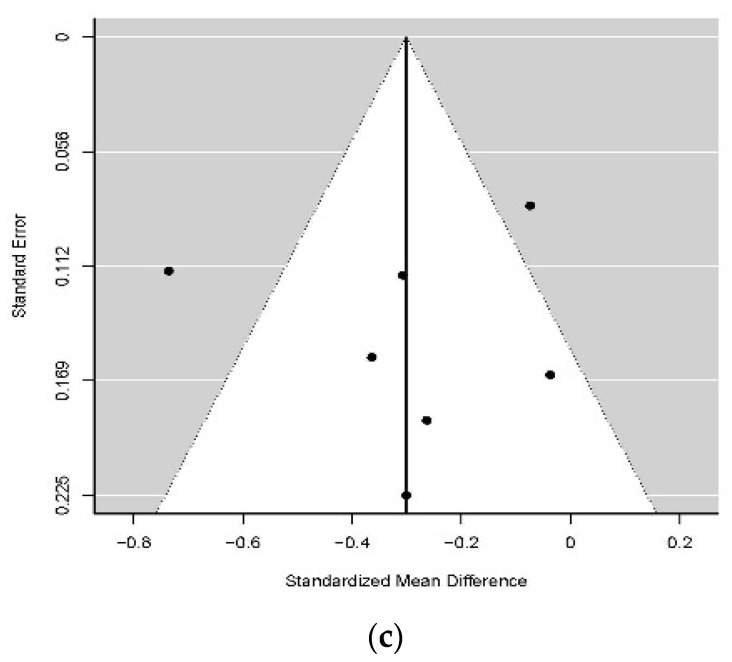
Funnel plot illustrations: (**a**) For randomized controlled trials on reducing stress; (**b**) for randomized controlled trials on reducing anxiety; (**c**) for randomized controlled trials on reducing depression. The fail-safe number *N* was calculated *(N =* 79), meaning that 79 studies on depression reduction were needed to nullify the significant effect at *p*  >  0.05.

Funnel plots were also created (Figure 5). Furthermore, Egger’s regression test and Egger’s and Begg–Mazumdar rank correlation tests for funnel plot asymmetry were utilized to evaluate publication predisposition in the meta-analysis [42] (p. 633). Publication bias was not found in the meta-analysis of studies with interventions for decreasing stress (*t* (4) = −0.56, *p* = 0.607). The Begg–Mazumdar rank correlation test for funnel plot asymmetry revealed that the funnel plot was symmetric (Kendall’s tau = −0.60, *p* = 0.136) (Figure 5a). The fail-safe *N* test was likewise conducted, and its computation utilizing the Rosenthal approach revealed that the fail-safe *N* = 98 was robust since “the fail-safe *N* is the number of nonsignificant studies necessary to make the outcome nonsignificant, and this number is robust when *N* > 5n + 10” [43] (p. 466).

As presented in Figure 5b, an almost symmetrical funnel plot of RCTs for diminishing anxiety indicated the absence of publication predisposition. The outcomes from Egger’s regression test (*t* (7) = −1.98, *p* = 0.088) and the Begg–Mazumdar rank correlation test for funnel plot asymmetry (Kendall’s tau = −0.33, *p* = 0.256) did not affirm that there was significant asymmetry in the funnel plot. This information shows that publication predisposition was not identified in the meta-analysis of RCTs for decreasing anxiety. The fail-safe *N* test revealed that the fail-safe number *N* was robust (*N* = 208) and that 208 examinations of anxiety decrease were expected to invalidate the significant impact at *p* > 0.05.

A funnel plot was created (Figure 5c) and Egger’s and Begg–Mazumdar tests were performed to assess publication bias in the meta-analysis of RCTs for reducing depression. However, publication bias was not found (*t* (5) = −0.37, *p* = 0.709; Kendall’s tau = 0.05, *p* = 1.000).

## 4. Discussion

The goal of the present meta-analysis was to scientifically test and demonstrate how effective internet-based psychological intervention programs are for reducing anxiety, depression, and stress in university students in the context of the COVID-19 pandemic. The evidence gathered in this meta-analysis parallels previous results reporting the effectiveness of psychological intervention programs targeting the most common psychological distress indicators among students and adolescents, namely stress, anxiety, and depression [44,45]. Our findings are in line with the results of the meta-analytical study by Tejada-Gallardo et al. [46], which indicated that multicomponent positive psychology interventions significantly reduce depression symptoms with small effects but do not affect anxiety. The results of our study also correlate with more recent previous results suggesting the small effectiveness of internet-based interventions targeting stress, anxiety, and depression [47,48,49]. In addition, in a meta-analysis of internet-based psychological interventions for mental health in university students comprising 48 studies, “small intervention effects were found on depression (*g* = 0.18, 95% CI [0.08, 0.27]), anxiety (*g* = 0.27, 95% CI [0.13, 0.40]), and stress (*g* = 0.20, 95% CI [0.02, 0.38])” [50] (p. 1). Our results are also in line with evidence from a previous meta-analysis of internet-based psychological interventions in non-college student populations [51] and university student populations [7]. Furthermore, in the meta-analysis by Heber et al. [51], internet-based psychological interventions “yielded a small effect size for stress (*d* = 0.43; 95% CI [0.1, 0.54]) but a lower small effect size for depression (*d* = 0.34; 95% CI [0.21, 0.48]) and anxiety (*d* = 0.32; 95% CI [0.17, 0.47]” (p. 1). Such a difference between the effects of internet-based psychological interventions may be explained by the differences in the baseline scores of the research participants. In the meta-analysis by Ma et al. [7], online guided self-help interventions had a pooled effect size for depression at posttest of “*g* = 0.46 (95% CI [0.28, 0.64], which was also considered small” [7] (p. 7).

The analysis of the six RCTs on internet-based mental interventions for stress decrease detailed that review (treatment) participants who received interventions had fundamentally lower stress than those in the control group at posttest; nonetheless, statistically significantly high heterogeneity was noticed. The meta-analysis of nine RCTs, which added to a pooled analysis of internet-based mental interventions for anxiety, revealed that there was a reduction in anxiety; however, this was not statistically significant, and exceptionally high heterogeneity was uncovered.

The analysis of the seven studies involving internet-based mental interventions for depression revealed that the participants in the review (treatment) group who received internet-based mental intervention had significantly lower depression than those in the control group at posttest; however, moderate heterogeneity was noticed. The moderate to high heterogeneity could be explained by contrasts in the fluctuations in the length of the sessions and duration of the interventions (the length of sessions varied in from 10 to 90 min, and the duration of the interventions ranged from three weeks to 12 weeks.) For instance, the subgroup examinations of interventional studies that Ma et al. [7] conducted revealed that intervention impacts were significant among the interventions of shorter (≤4 weeks), moderate (4–8 weeks), and greater lengths (≥8 weeks). The moderate to high heterogeneity here can be explained by contrasts in diagnostic tools (for example, just for anxiety estimation in our meta-analysis, six instruments were utilized: the BAI, BDI-II, OTAI, SHAI, PROMIS, and GAD-7. In summary, great heterogeneity has often been detailed in reviews on internet-based mental interventions for depression [52,53,54].

In our review, the impact size was viewed as small for internet-based mental interventions for stress (SMD: −0.36) and depression (SMD: −0.28). Furthermore, the findings on the information analysis of internet-based mental interventions for anxiety revealed that there was a decrease in anxiety (SMD: −0.65), but it was not statistically significant at 95% CI [−1.32, 0.02]. The collected discoveries on the impact size of these mental interventions were from just six RCTs on lessening stress, seven RCTs on lessening depression, and nine RCTs on lessening anxiety that were included in the meta-analysis, so they are generalized. Nevertheless, the quality of all RCTs in this meta-analysis was rated as high. Past meta-analyses that analyzed the effectiveness of mental interventions for lessening stress, anxiety, and depression among college undergraduates were exclusively of satisfactory quality (small samples, a modest number of sessions, or a brief intervention duration) [4,55]. Thus, we suggest that researchers maintain high standards of quality in their studies [49,50].

To summarize, the consequences of our review detail that internet-based mental interventions could lessen stress, anxiety, and depression among college undergraduates and graduates in the pandemic context. The present meta-analysis uncovered a few ramifications for training that people working in undergraduate health, such as counselors, might need to reflect on when offering internet-based resources to support their students. Regardless, college undergraduates were able to use college internet-based counseling resources during the quarantine period in addition to a good support instrument while waiting to see a qualified specialist.

A strength of the present study is that all reviews included in the meta-analysis were RCT examinations. The second strength is that it gave a comprehensive analysis of the viability of most current internet-based mental interventions for the psychological health issues of stress, anxiety, and depression. Therefore, we can claim that a major strength of this study is that with this meta-analysis, we gathered scientific evidence on how psychological internet-based interventions for reducing anxiety, depression, and stress in university students in the pandemic context are effective.

This meta-analysis has a few limitations. First, only English publications were included in the meta-analysis. Second, of the ten included studies, seven were conducted in America, two in Asia, one in Africa, and none in Europe; subsequently, further intervention studies in different nations could aid in generalizing our outcomes globally. Third, there was substantial variability in the number of sessions, which ranged from four to forty, so future researchers might need to consider comprising multiple (not just a few) sessions. Fourth, for all three groups of studies (studies on interventions to reduce stress, anxiety, and depression), statistically significant heterogeneity was observed, which was explained by the differences in variability in the number of sessions and the various diagnostic tools used. Statistically significant heterogeneity highlights a lack of consistency in the approaches used across RCTs. Therefore, more RCTs should be conducted to combine data quantitatively without statistically significant heterogeneity.

From a future research perspective, a meta-analysis of a present pandemic-focused study informed us of what types of internet-based interventions are most effective in reducing mental health issues (even though their effectiveness in reducing anxiety was not statistically significant). There may also be long-term mental health problems among university students, and this present pandemic-focused study showed us what types of internet-based intervention programs could be effective for reducing stress, depression, and anxiety among these students after the pandemic because “internet-based intervention programs may engender less stigma and be a more acceptable approach for meeting students’ mental health needs, thus reducing the treatment gap, and perhaps even providing a bridge to further treatment” [9] (p. 2) Future research could focus on people who are part of the environment of university students and are part of a vulnerable population during a quarantine (if it occurs again): parents, teachers, and others close to them. Future studies should also examine and compare the effects of in-person (face-to-face) versus internet-based psychological intervention programs on university students.

## 5. Conclusions

This meta-analysis showed that internet-based psychological interventions have significant effects on the reduction of stress and depression among university students during the COVID-19 pandemic. That is, internet-based psychological interventions may help to reduce mental health problems among university students, but further RCTs are needed to identify these interventions’ effectiveness for reducing anxiety. Additionally, more RCTs on reducing stress, anxiety, and depression among university students should be conducted using consistent approaches to avoid high heterogeneity. Overall, this study provides a basis for developing future internet-based programs addressing mental health problems among university students in the pandemic context.

## Figures and Tables

**Table 1:** Detailed descriptions of the reviewed articles.

Meta-Analysis ID	Author/Year	Sample Size	Age	Participant Details	Type of Intervention	Control Conditions	Number of Sessions or Duration of Intervention	Intervention Provider	Instruments	Quality Score
1	Chang et al. (2022)	*n* = 679 (E: 326, C: 352)	18 years or older	College undergraduate students	Brief online Isha Upa yoga modules for undergraduates’ mental health and wellbeing	Waitlist control	Modules (25 min) daily for 12 weeks	Isha Hatha yoga teacher, online	PSS-10 WEMWBS PHQ-4	6
2	Dorais and Gutierrez (2021)	*n* = 190 (E: 94, C: 96)	18 years or older	College undergraduate and graduate students	Centering meditation internet-based intervention	Waitlist control	Meditation for 10 min each morning and night (4 weeks)	College counselor, online	PSS-10	6
3	Krifa et al. (2021)	*n* = 366 (E: 183, C: 183)	Aged 18–30 years	Healthcare students	Internet-based positive psychology intervention	Waitlist control	88 sessions (8 weeks) of approximately 45 min each	Virtual instructors, online (videos)	DASS-21	6
4	Rackoff et al. (2022)	*n* = 585 (E: 301, C: 284)	18 years or older	College or university students	Self-help internet-based positive psychology and CBT intervention	Remote counseling services (as usual)	12 sessions (10 min each), 7 self-work sessions (30 min each) (4 weeks)	Virtual instructors, internet-based videos	DASS-21	5
5	Ritvo et al. (2021)	*n* = 154 (E: 76, C: 78)	18 years or older	College undergraduate students	MBI	Waitlist control	12 sessions of 20-min video conferences (8 weeks)	Moderator-psychologist, online	PSS-10, BAI, PHQ-9	6
6	Shehab (2021)	*n* = 80 (E: 40, C: 40)	18 years or older	College students	Breathing training program administered via a smartphone	Waitlist control	Two 10-min sessions per day for 5 days per week (44 weeks)	Study member and virtual instructor, breathing application	PSS-10, BAI, BDI-II	6
7	Alibak and Alibak (2021)	*n* = 48 (E1: 16, E2: 16, C: 16)	Aged 24–48 years	Graduate students	CBT (E1); Internet-based positive psychotherapy (E2)	Waitlist control	8 weeks, 1.5-h group therapy sessions (weekly)	Licensed psychologist, Zoom	OTAI	5
8	Shabahang et al. (2021)	*n* = 150 (E: 75, C: 75)	18 years or older	College students	Video-based CBT intervention	Waitlist control	Nine 15–20-min sessions (3 days per week for 3 weeks)	Experts, online (videos)	SHAI, ASI-3	6
9	Simonsson et al. (2021)	*n* = 177 (E: 88, C: 89)	Aged 18–24 years	University students	Online mindfulness intervention	Waitlist control	Weekly classes via Zoom of 90 min each (8 weeks)	Mindfulness teacher, Zoom	PROMIS	6
10	Sun et al. (2022)	*n* = 114 (E: 57, C: 57)	18 years or older	College students	Mindfulness-based mobile health intervention	Remote social support (as usual)	Weekly 1-h meetings (4 weeks)	Licensed psychologist, MBI teacher, Zoom	GAD-7, PHQ-9	5

Notes. E—study group. C—control group. CBT—cognitive-behavioral therapy. PSS-10—Perceived Stress Scale. WEMWBS—Warwick–Edinburg Mental Wellbeing Scale. PHQ-4—Patient Health Questionnaire for anxiety and depression measurement. DASS-21—Depression, Anxiety, and Stress Scales. MBI—Mindfulness-based intervention. PHQ-9—Patient Health Questionnaire for depression measurement. BAI—Beck Anxiety Inventory. BDI-II—Beck Depression Inventory-II. OTAI—Online Test Anxiety Inventory. SHAI—Short Health Anxiety Inventory. ASI-3—Anxiety Sensitivity Index-3 (not applicable in meta-analysis). PROMIS—Patient-Reported Outcome Measurement Information System (anxiety and depression scales). GAD-7—Generalized Anxiety Disorder Screener.

**Table 2:** Interventions for reducing the stress, anxiety, and depression of university students in the randomized controlled trials included in the meta-analysis.

			SG			CG		
Meta- Analysis ID	Authors	Year	*N*	Mean	SD	*N*	Mean	SD
Interventions for reducing stress among university students during COVID-19 in RCTs								
1	Chang et al.	2022	179	19.72	6.17	126	20.98	6.62
2	Dorais and Gutierrez	2021	61	17.52	5.01	89	19.49	5.66
3	Krifa et al.	2021	159	1.67	0.42	165	2.05	0.42
4	Rackoff et al.	2022	301	22.98	9.87	284	23.84	9.18
5	Ritvo et al.	2021	69	18.28	7.82	77	20.12	7.88
6	Shehab	2021	40	17.97	4.94	40	19.81	4.56
Interventions for reducing anxiety among university students during COVID-19 in RCTs								
1	Chang et al.	2022	179	4.86	1.75	126	4.60	1.84
3	Krifa et al.	2021	159	1.59	0.50	165	1.92	0.53
4	Rackoff et al.	2022	301	15.82	10.81	284	15.60	10.17
5	Ritvo et al.	2021	69	12.29	10.84	77	14.61	12.37
6	Shehab	2021	40	41.00	10.18	40	42.19	12.20
7	Alibak and Alibak	2021	14	14.75	1.48	14	33.31	7.10
8	Shabahang et al.	2021	75	30.61	4.01	75	37.25	3.32
9	Simonsson et al.	2021	79	9.81	3.54	86	11.70	3.7
10	Sun et al.	2022	57	6.08	3.99	57	6.13	4.26
Interventions for reducing depression among university students during COVID-19 in RCTs								
1	Chang et al.	2022	179	3.53	1.58	126	4.03	1.68
3	Krifa et al.	2021	159	1.53	0.51	165	1.91	0.52
4	Rackoff et al.	2022	301	19.90	12.32	284	20.80	11.98
5	Ritvo et al.	2021	69	7.81	6.41	77	8.05	6.30
6	Shehab	2021	40	11.15	9.21	40	13.95	9.22
9	Simonsson et al.	2021	79	8.81	3.75	86	10.23	4.0
10	Sun et al.	2022	57	6.42	3.76	57	7.63	5.24

Notes. SG—study group. CG—control group. *N*—number of participants during posttest. RCTs—randomized controlled trials.

**Table 3:** Results of the meta-analysis.

Group of Studies on Interventions in RCTs Included in Meta-Analysis	Studies	Q-Value	Heterogeneity *p*	*I^2^*	*p* of Meta-Analysis	SMD (95% CI) Random Effects	
For reducing stress	6	34.19	<0.001	81.99%	0.01	−0.36	(−0.61, −0.11)
For reducing anxiety	9	131.78	<0.001	97.86%	0.06	−0.65	(−1.32, 0.02)
For reducing depression	7	24.46	<0.001	71.46%	<0.001	−0.30	(−0.49, −0.11)

Notes. CI—confidence interval. SMD—standardized mean difference.

## Data Availability

Publicly available data were analyzed in this study.
